# Differential regulation of cell functions by CSD peptide subdomains

**DOI:** 10.1186/1465-9921-14-90

**Published:** 2013-09-08

**Authors:** Charles Reese, Shanice Dyer, Beth Perry, Michael Bonner, James Oates, Ann Hofbauer, William Sessa, Pascal Bernatchez, Richard P Visconti, Jing Zhang, Corey M Hatfield, Richard M Silver, Stanley Hoffman, Elena Tourkina

**Affiliations:** 1 Department of Medicine/Division of Rheumatology and Immunology, Medical University of South Carolina, 171 Ashley Avenue, Charleston, SC 29425, USA; 2 Department of Regenerative Medicine and Cell Biology, Medical University of South Carolina, 171 Ashley Avenue, Charleston, SC 29425, USA; 3 Department of Pharmacology and Vascular Biology and Therapeutics Program, Yale University School of Medicine, New Haven, CT 06520, USA; 4 Department of Anesthesiology, The James Hogg Research Centre, Heart and Lung Institute at St. Paul’s Hospital, Pharmacology and Therapeutics, University of British Columbia, St. Paul’s Hospital, Vancouver, British Columbia, Canada; 5 Division of Rheumatology and Immunology, Department of Medicine, Medical University of South Carolina, 96 Jonathan Lucas Street, Suite 912 MSC 637, Charleston, SC 29425, USA

**Keywords:** Caveolin-1, Monocytes, Fibrocytes, Fibroblasts, Scleroderma (SSc), Migration, TGFβ

## Abstract

**Background:**

In fibrotic lung diseases, expression of caveolin-1 is decreased in fibroblasts and monocytes. The effects of this deficiency are reversed by treating cells or animals with the caveolin-1 scaffolding domain peptide (CSD, amino acids 82–101 of caveolin-1) which compensates for the lack of caveolin-1. Here we compare the function of CSD subdomains (Cav-A, Cav-B, Cav-C, Cav-AB, and Cav-BC) and mutated versions of CSD (F92A and T90A/T91A/F92A).

**Methods:**

Migration toward the chemokine CXCL12 and the associated expression of F-actin, CXCR4, and pSmad 2/3 were studied in monocytes from healthy donors and SSc patients. Fibrocyte differentiation was studied using PBMC from healthy donors and SSc patients. Collagen I secretion and signaling were studied in fibroblasts derived from the lung tissue of healthy subjects and SSc patients.

**Results:**

Cav-BC and CSD at concentrations as low as 0.01 μM inhibited the hypermigration of SSc monocytes and TGFβ-activated Normal monocytes and the differentiation into fibrocytes of SSc and Normal monocytes. While CSD also inhibited the migration of poorly migrating Normal monocytes, Cav-A (and other subdomains to a lesser extent) promoted the migration of Normal monocytes while inhibiting the hypermigration of TGFβ-activated Normal monocytes. The effects of versions of CSD on migration may be mediated in part via their effects on CXCR4, F-actin, and pSmad 2/3 expression. Cav-BC was as effective as CSD in inhibiting fibroblast collagen I and ASMA expression and MEK/ERK signaling. Cav-C and Cav-AB also inhibited collagen I expression, but in many cases did not affect ASMA or MEK/ERK. Cav-A increased collagen I expression in scleroderma lung fibroblasts. Full effects on fibroblasts of versions of CSD required 5 μM peptide.

**Conclusions:**

Cav-BC retains most of the anti-fibrotic functions of CSD; Cav-A exhibits certain pro-fibrotic functions. Results obtained with subdomains and mutated versions of CSD further suggest that the critical functional residues in CSD depend on the cell type and readout being studied. Monocytes may be more sensitive to versions of CSD than fibroblasts and endothelial cells because the baseline level of caveolin-1 in monocytes is much lower than in these other cell types.

## Background

Caveolin-1, a protein associated with plasma membrane invaginations known as caveolae and with other cellular membranes, is a promising therapeutic target in ILDs. We and others have shown that caveolin-1 is deficient in the lung tissue of SSc and IPF patients and in cells isolated from the lung tissue and blood of these patients including fibroblasts, monocytes, and neutrophils [1-3]. Similarly, caveolin-1 is also deficient in mice in which ILD has been induced with bleomycin or irradiation [2,3].

Caveolin-1 binds to and thereby inhibits the function of kinases in several major families including PKC, MAPK, Src, and G protein [4-7] and regulates signaling and cell functions induced by the major pro-fibrotic cytokine, TGFβ [1,8,9]. The effects of caveolin-1 deficiency in cells and in animals can be reversed either by using adenovirus encoding full-length caveolin-1 or using the caveolin-1 scaffolding domain peptide (CSD; amino acids 82–101 of caveolin-1) [1,2]. When CSD is synthesized in fusion with the Antennapedia Internalization Sequence, it can enter cells and inhibit kinases just like full-length caveolin-1 [10,11]. CSD was reported [4] to bind to target kinases through consensus sequences (ΦXΦXXXXΦ and ΦXXXXΦXXΦ) where Φ stands for any of the aromatic amino acids (F, W, or Y) and X stands for any amino acid. Later studies suggested that the initial definition of the consensus sequences was overly stringent and that the consensus sequences for caveolin binding domains (CBDs) are ΦXZXXXXΦ and ZXXXXΦXXZ where Z stands for F, W, Y, I, V, or L [12].

Given the large number of signaling molecules that contain CBDs and the heterogeneity of the primary sequences of these CBDs, it is extremely likely that subdomains of CSD will differ from each other and from CSD in their ability to regulate the activity of these kinases and therefore will have distinctive effects on cell behavior. Indeed, previous studies on CSD subdomains have given distinct results depending on the peptide being studied. For example, in experiments using endothelial cells, amino acids 89–95, 82–95, and 89–101 all inhibited eNOS production and it was therefore concluded that 89–95 was the key sequence involved in this process [11,13]. In contrast, 86–101, but not 88–101, inhibited the activity of PKC isoforms purified from transfected H5 insect cells [5]. Similarly, CSD, but not 84–92 or 93–101, inhibited the activity of MEK and ERK purified from bacterial extracts [14].

In order to identify CSD subdomains that may be more useful than full-length CSD in treating human diseases, here we have compared the ability of CSD and several subdomains and mutated versions (each attached to the Antennapedia Internalization Sequence) to reverse effects associated with low caveolin-1 on the behavior of monocytes (migration toward CXCL12; expression of CXCR4 and F-actin and Smad 2/3 activation; differentiation into fibrocytes) and fibroblasts (collagen I and ASMA expression, MEK/ERK activation). For these experiments, cells were isolated from both normal subjects and scleroderma patients. Overall, the Cav-BC peptide (amino acids 89–101) was as effective as, and sometimes more effective than, full-length CSD. Interestingly, the Cav-A peptide (amino acids 82–88) in some cases exacerbated effects associated with low caveolin-1. While not surprising, it is noteworthy that the patterns of relative activity that we observed with CSD subdomains and with mutated versions of CSD differed from those obtained in a study of CSD regulation of eNOS-mediated NO release in endothelial cells [11].

## Methods

### Subjects for monocyte studies

Under a protocol approved by the Institutional Review Board for Human Research for a Rheumatology Research Repository, patients with SSc-ILD were recruited from the Scleroderma Clinic at the Medical University of South Carolina (MUSC). All patients fulfilled the American College of Rheumatology (formerly the American Rheumatism Association, ARA) criteria for SSc [15] and had evidence of SSc-ILD as previously defined [16]. Demographic data for SSc patients and normal healthy donors are summarized in Table 1.

**Table 1:** Clinical features of SSc patients involved in monocyte experiments

**Race/Smoking**	**Gender**	**Patients**	**Controls**
Caucasian	M	2	9
Caucasian	F	7	12
African-American	M	3	2
African-American	F	3	9
Asian	F	0	1
Smoker		0	2
Former Smoker		2	3
Age: Mean ± SD (range)	Patients	52.3 ± 12.8 (27–79)	
	Controls	43.0 ± 11.9 (18–64)	
Disease	Limited Cutaneous	3	
	Diffuse Cutaneous	9	
	Overlap	3	
Disease duration: Mean ± SD (range), yr		4.3 ± 2.8 (1-10)	
Pulmonary Involvement (ILD)		15/15 (100%)	
Pulmonary HTN		3/15 (20%)	
GI Involvement		15/15 (100%)	
Cardiac Involvement		2/15 (13.3%)	
Renal Involvement		1/15 (6.7%)	
Autoantibodies:	ANA+	15/15 (100%)	
	Scl-70+	6/15 (40%)	
	Anti-centromere	1/15 (6.7%)	

### Monocyte isolation

Monocytes were isolated by standard methods [16,17]. Following centrifugation on density 1.083 Histopaque cushions, monocytes were enriched by immunodepletion using a Dynal Monocyte Negative Isolation Kit (Invitrogen, Carlsbad, CA) resulting in a cell population about 95% Mac-1+ monocytes.

### Monocyte migration

Assays were performed as previously described [17]. Briefly, CXCL12 (100 ng/ml in RPMI 1640 with 1% BSA) was placed into the lower wells of Neuro Probe Multiwell Chemotaxis Chambers (Neuro Probe, Gaithersburg, MD) fitted with 5-μm pore size polycarbonate filters. 25 μl of cell suspension (1 × 10^6^ cells/ml) with or without TGFβ pretreatment (45 min, 10 ng/ml in RPMI 1640 with 1% BSA) was placed in the upper wells. Peptides were added to cells before they were placed in the upper chamber. After incubation for 2.5 h at 37°C in a 5% CO_2_ incubator, filters were removed, fixed, and stained with DAPI (Invitrogen, Carlsbad, CA). Cells on the underside of the membrane were photographed and counted in six high power fields per filter.

### F-actin, CXCR4, and pSmad 2/3 levels

Were evaluated by immunocytochemistry in monocytes that were isolated as described above, cultured overnight in 6-well plates (1 × 10^5^ cells per well) on coverslips in RPMI 1640/ 20% FCS, and sequentially treated with RPMI 1640/ 1% BSA with or without 10 ng/ml TGFβ, then with the same medium containing 5 μM of the indicated peptides as previously described [16]. Cells were then fixed, labeled with FITC phalloidin (Sigma-Aldrich), rabbit anti-CXCR4 (Santa Cruz Biotechnology sc-9046), rabbit anti pSmad 2/3 (Cell Signaling 3102) Alexa Fluor® 555-conjugated secondary antibodies (Invitrogen), and counterstained with the nuclear stain DAPI. Staining was quantified in arbitrary units by image analysis of ten cells in each category in terms of the average fluorescence intensity ± s.e.m.

### Smad western blots

Monocytes (2 × 10^6^ cells per well) incubated as described above for pSmad 2/3 immunocytochemistry were next washed twice with PBS, then extracted with SDS-PAGE sample buffer. Smad 2/3 and pSmad 2/3 levels were determined by Western blotting using rabbit anti-Smad 2/3 (Cell Signaling 3102), rabbit anti-pSmad 2/3 (Cell Signaling 3101), and mouse monoclonal anti-GAPDH (EMD Millipore MAB374, clone 6C5) as a loading control.

### Monocyte to fibrocyte differentiation

Total PBMC from 40 ml of peripheral blood were plated in eight wells of fibronectin-coated six-well plates in DMEM/ 20% FCS with supplements for 12 days [18]. Medium was changed on day 5. Peptides were added on day 2 and again on day 5 after the medium was changed. Specific peptides used are described in the Figure Legends. Images were acquired on day 12 and fibrocytes were quantified in terms of elongated cells per 10 × field, all of which were collagen I+. For immunocytochemistry, the same methods were used except that the wells contained coverslips.

### Subjects for fibroblast studies

Fibroblasts were derived from lung tissue obtained at autopsy from SSc patients (SLF) and from normal subjects (NLF) and cultured as previously described [7]. Cells were used in passages 2–4. SSc lung tissue was obtained from the Division of Pathology and Laboratory Medicine at the Medical University of South Carolina (MUSC). These SSc patients fulfilled the criteria of American College of Rheumatology for the diagnosis of SSc with lung involvement. Normal human lung tissue was obtained from the Brain and Tissue Bank for Developmental Disorders (Baltimore, MD) or from the National Disease Research Interchange (Philadelphia, PA). The study was approved by the Institutional Review Board for Human Subject Research at the MUSC as non-human research. The demographics of these subjects are provided in Table 2.

**Table 2:** Clinical features of autopsy samples used in fibroblast experiments

**Race/Smoking**	**Gender**	**Patients**	**Controls**
Caucasian	M	0	1
Caucasian	F	4	2
African-American	M	0	0
African-American	F	1	0
Smoker		1	0
Former Smoker		1	1
Age: Mean ± SD (range)	Patients	46.6 ± 17.4 (21-62)	
	Controls	56.6 ± 8.5 (46-65)	
Disease:	Limited Cutaneous	4	
	Diffuse Cutaneous	1	
	Overlap	0	
Pulmonary Involvement (ILD)		5/5 (100%)	
GI Involvement		4/5 (80%)	
Cardiac Involvement		4/5 (80%)	
Renal Involvement		1/5 (20%)	

### Fibroblast collagen I secretion and signaling

As previously described [8], aliquots of culture medium or of cell layer, representing material derived from the same number of cells, was probed on Western blots using the following primary antibodies and appropriate secondary antibodies: rabbit antibodies against ERK 1/2 (9102), pERK 1/2 (9106), MEK 1/2 (9122), and pMEK 1/2 (9121) from Cell Signaling (Beverly, MA); mouse monoclonal anti-ASMA (A2547, clone 1A4) from Sigma (Saint Louis, MO), and mouse monoclonal anti-actin (MAB1501) from Millipore (Temecula, CA) and goat anti-collagen I (AB758) from Millipore.

### Fibroblast immunocytochemistry

NLF and SLF were cultured on coverslips and stained as previously described [2,7] using the ASMA and pERK antibodies indicated above and appropriate secondary antibodies tagged with Alexa Fluor® 488. Nuclei were stained with DAPI. Images were acquired using a Zeiss 510SML Laser Confocal Microscope (excitation S490/20, emission D528/38) fitted with an oil-immersion objective (40 × /1.4).

### Peptides

To compare the activity of CSD (amino acids 82–101 of caveolin-1) to its subdomains, CSD and five subdomains named by Bernatchez et al. [11] [ Cav-A (aa 82–88), Cav-B (aa 89–95), Cav-C (aa 96–101), Cav-AB (aa 82–95) and Cav-BC (aa 89–101)] were synthesized in fusion with the Antennapedia Internalization Sequence. We routinely refer to these fusions simply as CSD, Cav-A, Cav-B, Cav-C, Cav-AB, and Cav-BC. The Antennapedia Internalization Sequence alone was routinely used as Control peptide, when tested scrambled CSD gave similar results to the Antennapedia Internalization Sequence alone. In addition, mutated CSD peptides in which F92 was converted to A and in which T90, T91, and F92 were all converted to A (referred to respectively as 92A and 90-92A) were synthesized. CSD, Cav-A, Cav-B, Cav-C, Cav-AB, Cav-BC, 92A, 90-92A, and control peptides were dissolved at 10 mM in 100% DMSO, diluted 10-fold with water as previously described, and further diluted as appropriate for each experiment [2,11,16,17].

### Statistical analyses

Immunoreactive bands were quantified by densitometry using ImageJ 1.32 NIH software. Raw densitometric data were processed and analyzed using Prism 3.0 (GraphPad Software Inc.). Student’s t-test was used to evaluate data. In all Figures, *** indicates p < 0.001, ** indicates p < 0.01, and * indicates p < 0.05.

## Results

### CSD and its subdomains differ in their ability to inhibit the migration of normal and SSc monocytes

**Figure 1:**
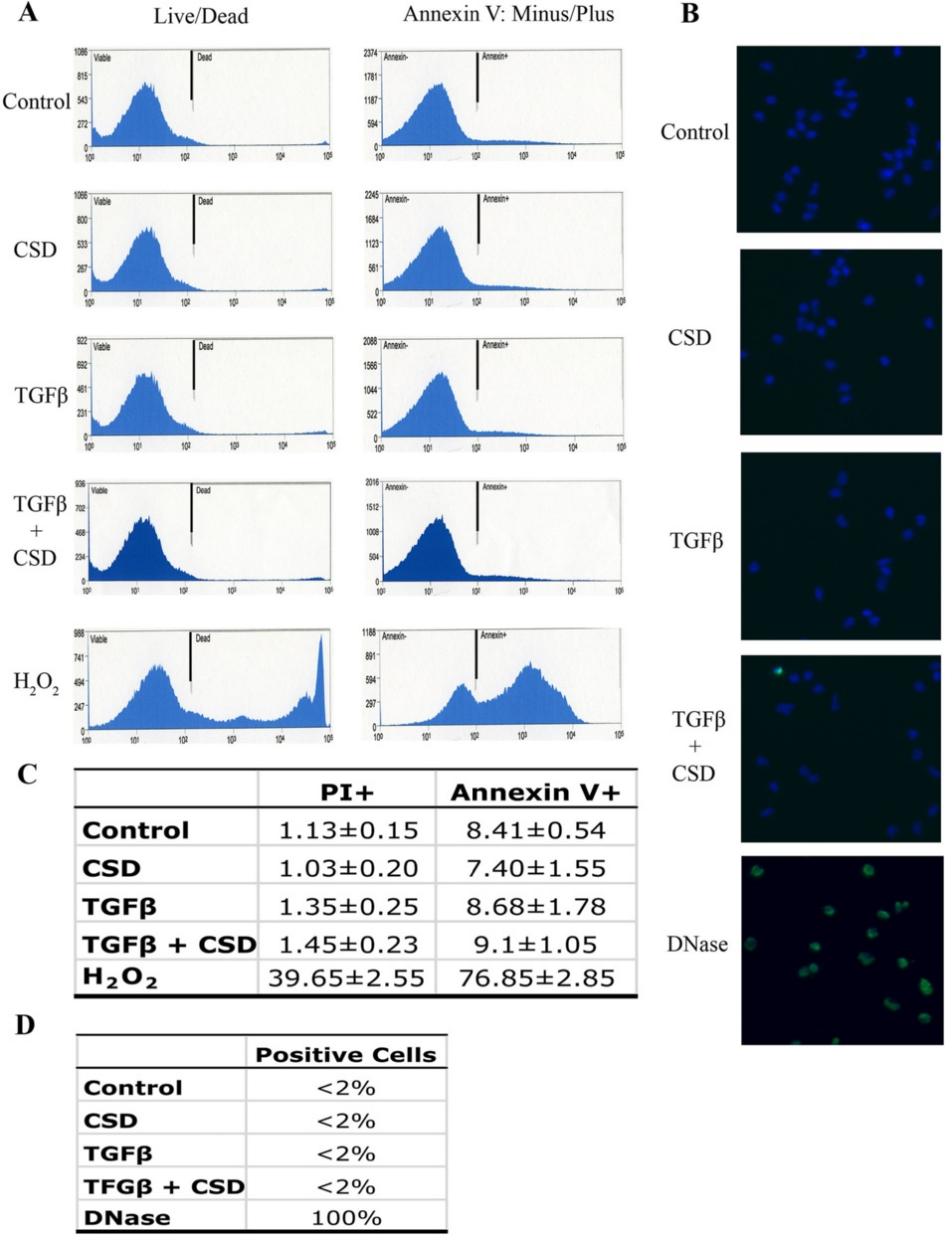
**Effects of CSD and TGFβ on monocyte migration do not involve cell death/apoptosis. (A)** Cell death/apoptosis was evaluated by flow cytometry using Molecular Probes kit V13241. Briefly, cells were treated with CSD, TGFβ, or both reagents (see Methods). Cells were then incubated with propidium iodide (PI) and fluorescent annexin V and analyzed by flow cytometry. Gates were set using unstained cells. A typical experiment is shown in which live cells (PI-negative) were gated from dead cells (PI-positive) indicated as Live/Dead. Live cells were further gated into apoptotic cells (annexin V-positive) and non-apoptotic cells (annexin V-negative) indicated as Annexin V: Minus/Plus. To validate the assay, the indicated cells were treated with 20 mM H_2_O_2_ (a known inducer of cell death/apoptosis). **(B)** Apoptosis was evaluated by TUNEL labeling using the In Situ Cell Death Detection Kit, Fluorescein (Product No. 11684795910, Roche, Indianapolis, IN). Briefly, cells on coverslips were treated with CSD, TGFβ, or both reagents (see Methods); fixed; permeabilized; DNA strand breaks fluorescently labeled using TUNEL reagent; and imaged by fluorescent microscopy. A typical experiment is shown in which essentially no cells were labeled except when permeabilized cells were treated with DNase prior to TUNEL labeling. Nuclei were counter-stained using DAPI. **(C)** Flow cytometry quantification. Average values ± s.e.m. are presented summarizing the results of four independent experiments performed using cells from different subjects. PI + indicates the percentage of dead PI-positive cells; Annexin V + indicates the percentage of the PI-negative cell population that is annexin V-positive (i.e. apoptotic). **(D)** TUNEL quantification. Three independent experiments were performed using cells from different subjects. The percentage of fluorescent, TUNEL-positive cells was always < 2% except when DNA strand breaks were generated using DNase.

We previously demonstrated that SSc monocytes and TGFβ-treated normal monocytes are hypermigratory toward the CXCR4 ligand CXCL12 and that this migration is inhibited by CSD [17]. To evaluate the possible role of regulation of cell death and/or apoptosis by TGFβ or CSD in their effects on migration, we studied cell death (measured by propidium iodide staining) and apoptosis (measured in terms of cell surface annexin V labeling). We found that neither TGFβ nor CSD affected either cell death or apoptosis even though in a positive control experiment validating the method, H_2_O_2_ (a known inducer of cell death and apoptosis) did have the expected effects (Figure 1A,C). To further validate these observations, apoptosis was also evaluated by TUNEL labeling. Again, essentially no apoptosis was observed in control cells or in cells treated with CSD, TGFβ, or both reagents even though in a positive control TUNEL labeling was observed (Figure 1B,D). These observations strongly suggest that the effects of CSD and TGFβ on migration are not mediated via effects on cell death or apoptosis.

**Figure 2:**
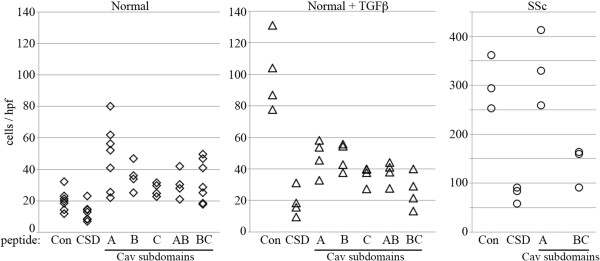
**Effects of CSD and its subdomains on Normal and SSc monocyte migration in vitro.** Normal monocytes **(A)** were isolated from healthy donors, SSc monocytes **(C)** were isolated from SSc patients. The migration of these cells and Normal monocytes pretreated with TGFβ **(B)** toward CXCL12 was quantified in the presence of 5 μM of CSD or the indicated subdomains of CSD as described in the Methods. The Antennapedia Internalization Sequence alone was routinely used as the Control peptide; when tested scrambled CSD attached to the Antennapedia Internalization Sequence gave similar results. Each symbol represents the results obtained with cells from an individual donor.

When we examined the effect of CSD subdomains on the migration of normal monocytes, we found that while all peptides used at the standard concentration of 5 μM [2,10,11,16,17] inhibited TGFβ-induced hypermigration, only CSD inhibited the low level of migration observed in the absence of TGFβ (Figure 2). Indeed, other peptides, especially Cav-A, appeared to increase this background migration (Figure 2, Table 3). While the hypermigration of SSc monocytes was similar to the hypermigration of TGFβ-treated monocytes in that both were inhibited by CSD and Cav-BC, they differed dramatically in their response to Cav-A which inhibited TGFβ-induced monocyte hypermigration but slightly increased SSc monocyte hypermigration (Figure 2, Table 3). Cav-B, Cav-C, and Cav-AB also inhibited TGFβ-induced monocyte hypermigration, although not as effectively as CSD and Cav-BC (Figure 2, Table 3).

**Table 3:** Quantification and statistical significance of monocyte migration and pSmad 2/3 staining data

**Monocytes**	**Peptide**	**Migration**	**Statistical significance**	**pSmad 2/3**	**Statistical significance**
Normal	Control	20.1 ± 2.5		26.3 ± 1.1	
Normal	CSD	12.6 ± 2.1	p < 0.05 vs Normal/Control	16.4 ± 0.5	p < 0.01 vs Normal/Control
Normal	Cav-A	48.4 ± 7.8	p < 0.01 vs Normal/Control	31.0 ± 1.1	p < 0.05 vs Normal/Control
Normal	Cav-B	35.4 ± 4.5	p < 0.01 vs Normal/Control	16.6 ± 1.6	p < 0.01 vs Normal/Control
Normal	Cav-C	27.1 ± 2.1		19.1 ± 1.5	p < 0.01 vs Normal/Control
Normal	Cav-AB	37.8 ± 8.7	p < 0.05 vs Normal/Control	26.8 ± 1.4	
Normal	Cav-BC	32.6 ± 5.1	p < 0.05 vs Normal/Control	18.4 ± 1.2	p < 0.01 vs Normal/Control
Normal + TGFβ	Control	99.9 ± 11.7	p < 0.001 vs Normal/Control	43.1 ± 0.9	p < 0.01 vs Normal/Control
Normal + TGFβ	CSD	18.7 ± 4.5	p < 0.01 vs Normal + TGFβ/Control	21.2 ± 1.2	p < 0.01 vs Normal + TGFβ/Control
Normal + TGFβ	Cav-A	47.4 ± 5.5	p < 0.01 vs Normal + TGFβ/Control	37.7 ± 1.2	
Normal + TGFβ	Cav-B	47.6 ± 4.4	p < 0.01 vs Normal + TGFβ/Control	40.7 ± 1.2	
Normal + TGFβ	Cav-C	36.2 ± 3.0	p < 0.01 vs Normal + TGFβ/Control	43.0 ± 1.1	
Normal + TGFβ	Cav-AB	37.7 ± 3.5	p < 0.01 vs Normal + TGFβ/Control	39.0 ± 1.5	
Normal + TGFβ	Cav-BC	25.9 ± 5.7	p < 0.01 vs Normal + TGFβ/Control	18.7 ± 1.4	p < 0.01 vs Normal + TGFβ/Control
SSc	Control	303 ± 32	p < 0.001 vs Normal/Control	54.1 ± 1.0	p < 0.01 vs Normal/Control
SSc	CSD	77 ± 10	p < 0.01 vs SSc/Control	37.6 ± 0.7	p < 0.01 vs SSc/Control
SSc	Cav-A	334 ± 44		49.1 ± 3.1	
SSc	Cav-B	Not Done		48.4 ± 1.5	
SSc	Cav-C	Not Done		49.3 ± 1.2	
SSc	Cav-AB	Not Done		46.1 ± 3.0	
SSc	Cav-BC	138 ± 24	p < 0.02 vs SSc/Control	36.6 ± 1.4	p < 0.01 vs SSc/Control

**Figure 3:**
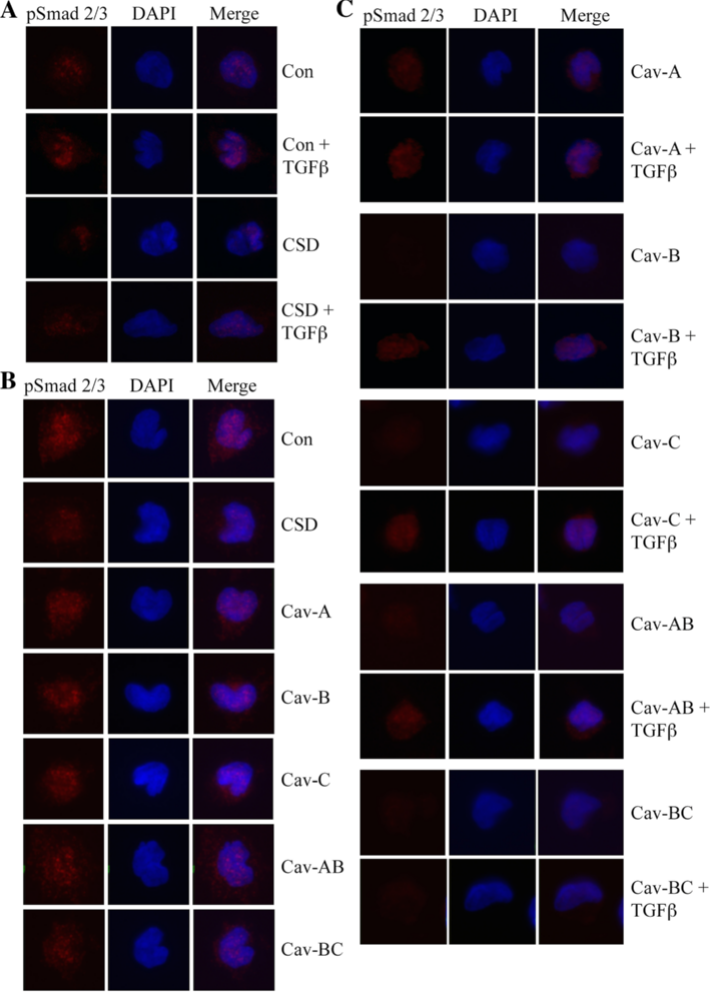
**Effects of CSD and its subdomains on pSmad 2/3 expression in normal, TGFβ-treated, and SSc monocytes.** As described in the Methods, monocytes were isolated, plated on coverslips, treated with the indicated reagents (TGFβ, CSD, or its subdomains), fixed, and stained for pSmad 2/3 and with the nuclear stain DAPI. Representative images are shown selected from 20 to 60 cells observed from four donors in each category. **(A)** Normal monocytes treated with TGFβ and CSD; **(B)** SSc monocytes treated with CSD and its subdomains; **(C)** Normal monocytes treated with TGFβ and CSD subdomains. The Antennapedia Internalization Sequence alone was routinely used as the Control peptide; when tested scrambled CSD attached to the Antennapedia Internalization Sequence gave similar results.

Data from Figure 2 (migration) and Figure 3 (image analyses of pSmad 2/3 staining) are presented in terms of average value ± s.e.m. The statistical significance of the indicated comparisons were determined using Students’ t test.

Because TGFβ enhanced monocyte migration and this effect was inhibited by CSD and each subdomain, we examined the effect of CSD and its subdomains on canonical TGFβ signaling via the activation of Smad 2/3. Immunocytochemical analyses of activated Smad 2/3 (i.e. pSmad 2/3) levels in monocytes showed a strong correlation between ability to migrate and pSmad 2/3 levels under several conditions (Figure 3, quantified in Table 3). For example, the enhanced ability of TGFβ-treated normal monocytes and SSc monocytes to migrate is accompanied by an increase in pSmad 2/3 levels. The abilities of CSD and Cav-BC to strongly inhibit the migration of TGFβ-treated normal monocytes and SSc monocytes are accompanied by a major decrease in pSmad 2/3 levels. The activation of Normal monocyte migration by Cav-A is accompanied by an increase in pSmad 2/3 level. On the other hand, various CSD subdomains enhance the migration of Normal monocytes or inhibit the migration of TGFβ-treated normal monocytes while having no effect or an opposite effect on pSmad 2/3 level. In summary, these studies demonstrate that while CSD and its subdomains can affect TGFβ signaling through pSmad 2/3, it is likely that both their effects on TGFβ signaling and their well-known effects on other signaling cascades together regulate monocyte migration.

**Figure 4:**
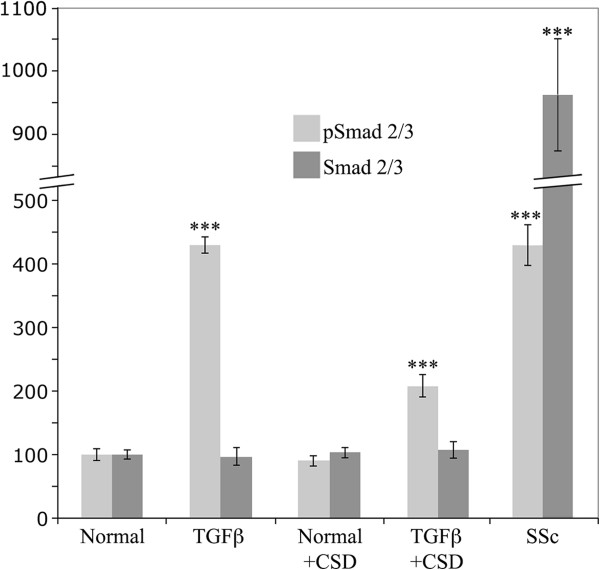
**Smad 2/3 expression and activation in monocytes.** The indicated monocytes were isolated and treated with CSD (or control peptide) as described in the Methods (Smad Western blots). TGFβ indicates TGFβ-treated Normal monocytes. Smad 2/3 expression and activation (i.e. pSmad 2/3 levels) were evaluated by Western blotting (50 μg total protein per lane). The data shown are the average ± s.e.m. in arbitrary units of the densitometric quantification of three independent experiments with cells from different subjects. The levels of Smad 2/3 and pSmad 2/3 in Normal monocytes treated with control peptide (normalized against the GAPDH loading control) were set to 100 arbitrary units. Indications of statistical significance for TGFβ-treated Normal monocytes and SSc monocytes are versus Normal monocytes. The indication of statistical significance for TGFβ-treated Normal monocytes treated with CSD is versus TGFβ-treated Normal monocytes treated with control peptide.

To further validate these results, select experiments were repeated and analyzed by Western blot (Figure 4). In accord with the immunocytochemical data, the pSmad 2/3 level was much higher in SSc monocytes than in Normal monocytes. Interestingly, the Smad 2/3 level was increased to an even greater extent than the pSmad 2/3 level in SSc monocytes (p < 0.001), suggesting that the increase in pSmad 2/3 is driven by the increase in Smad 2/3. Also in accord with the immunocytochemical data, the pSmad 2/3 level in Normal monocytes was increased by TGFβ treatment and this increase was substantially blocked by CSD. In this case, the increase in pSmad 2/3 level occurred in the absence of any change in Smad 2/3 level.

### CSD and its subdomains differ in their effects on CXCR4 expression and F-actin staining in normal and SSc monocytes

**Figure 5:**
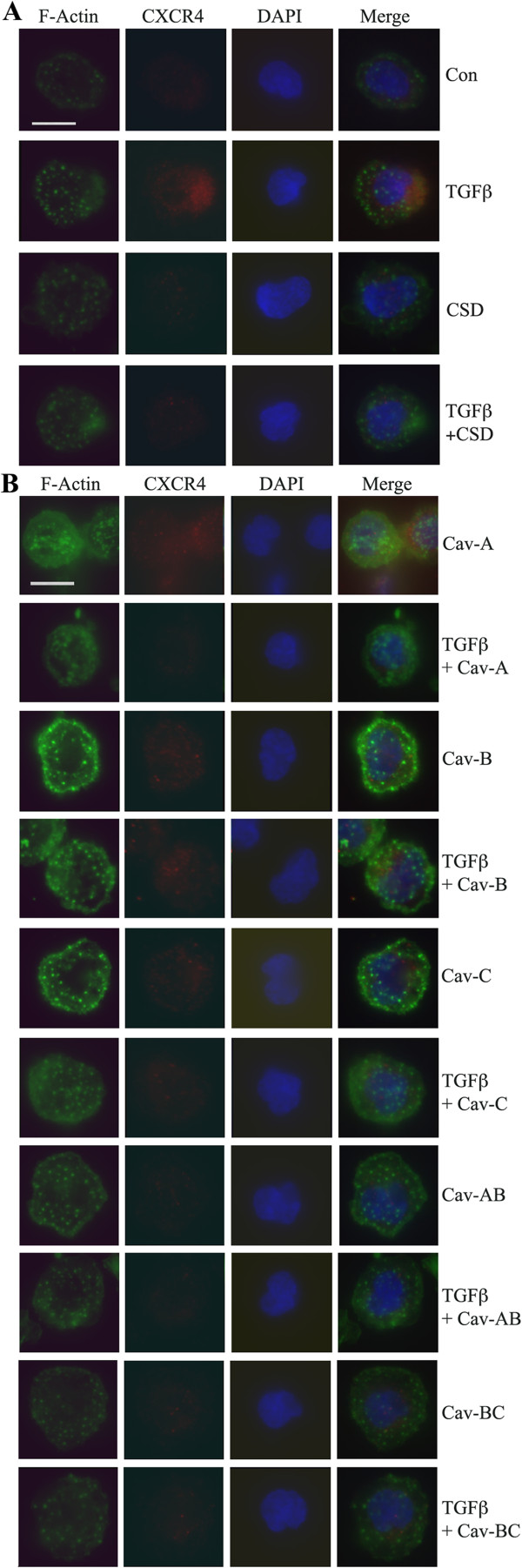
**Effects of TGFβ, CSD, and its subdomains on F-actin and CXCR4 expression in normal monocytes.** As described in the Methods, normal monocytes were isolated, plated on coverslips, treated with the indicated reagents (TGFβ, CSD, or its subdomains), fixed, and stained for CXCR4 and F-actin and with the nuclear stain DAPI. Representative images are shown selected from 20 to 60 cells observed from four donors in each category. **(A)** Normal monocytes treated with TGFβ and CSD; **(B)** Normal monocytes treated with TGFβ and CSD subdomains. The Antennapedia Internalization Sequence alone was routinely used as the Control peptide; when tested scrambled CSD attached to the Antennapedia Internalization Sequence gave similar results.

To evaluate the mechanisms underlying the differential effects of CSD and its subdomains on migration, we compared the effects of these peptides on the expression of CXCR4 and F-actin. Treatment of monocytes from healthy donors with TGFβ significantly increased CXCR4 expression and this increase was completely inhibited by CSD (Figure 5A, Table 3). In contrast, TGFβ only slightly increased F-actin staining (Figure 5A) and this increase was not fully reversed by CSD. When other peptides were examined (Figure 5B, Table 3), the results were more complicated because, in many cases, the peptide affected CXCR4 and F-actin expression in the absence of TGFβ. Cav-A, Cav-B, Cav-C, and to a lesser extent Cav-AB promoted F-actin staining and this effect was decreased by TGFβ. Cav-BC, like CSD, had little effect on F-actin staining. Cav-A, and to a lesser extent Cav-B and Cav-C, increased CXCR4 expression in normal cells while each peptide inhibited CXCR4 expression similarly in TGFβ-treated cells (Figure 5B, Table 3).

**Figure 6:**
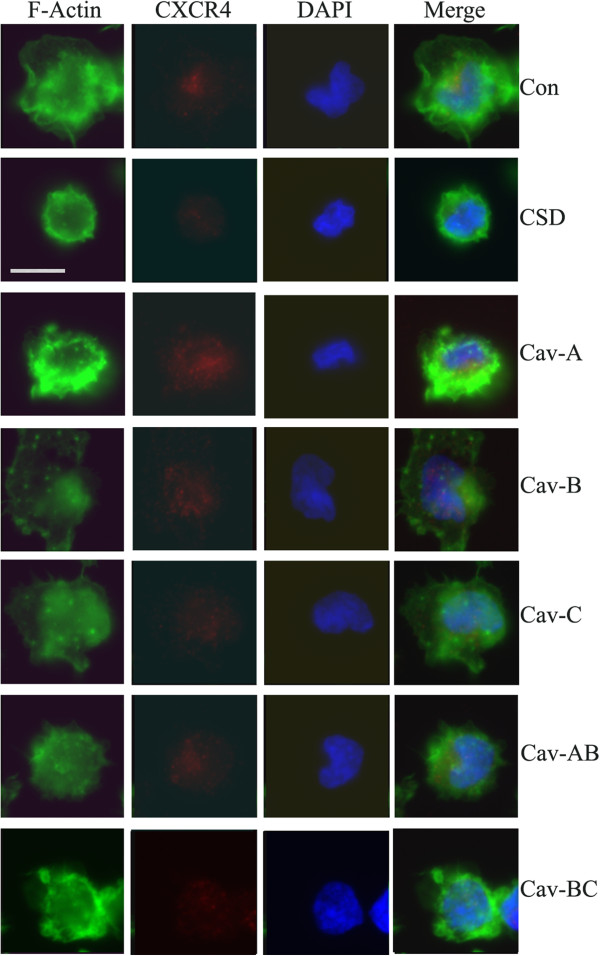
**Effects of CSD and its subdomains on F-actin and CXCR4 expression in SSc monocytes.** As described in the Methods, SSc monocytes were isolated, plated on coverslips, treated with the indicated reagents (CSD or its subdomains), fixed, and stained for CXCR4 and F-actin and with the nuclear stain DAPI. The Antennapedia Internalization Sequence alone was routinely used as the Control peptide; when tested scrambled CSD attached to the Antennapedia Internalization Sequence gave similar results. Representative images are shown selected from 20 to 60 cells observed from four donors in each category.

Experiments using SSc monocytes revealed a much higher level of CXCR4 expression [16] and F-actin staining than in normal monocytes and a somewhat different pattern of sensitivity to caveolin-1 peptides (Figure 6, Table 3). As previously shown, CSD inhibited CXCR4 expression. Cav-BC also inhibited CXCR4 expression as did Cav-B, Cav-C, and Cav-AB to a lesser extent. In contrast, as in healthy monocytes, Cav-A increased CXCR4 expression (Figure 6). CSD treatment also inhibited F-actin staining. In addition, the effect of CSD on the cytoskeleton results in smaller cells. Cav-B and Cav-AB and to a lesser extent Cav-C and Cav-BC somewhat inhibited F-actin staining.

In summary, for each type of monocyte (Normal, Normal + TGFβ, SSc) the expression of CXCR4 and F-actin, particularly CXCR4, are predictive of the level of migration. Nevertheless, CXCR4 and F-actin levels are not sufficient to explain the different ability of each type of monocyte to migrate strongly suggesting that CXCR4 and F-actin levels are not the only differences between Normal, Normal + TGFβ, and SSc monocytes.

### CSD and its subdomains differ in their ability to inhibit monocyte to fibrocyte differentiation in vitro

**Figure 7:**
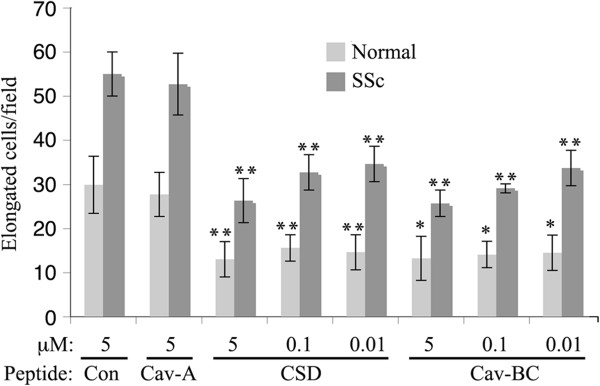
**Different effects of CSD and its subdomains on fibrocyte differentiation.** PBMC were incubated for fibrocyte differentiation in vitro as described in the Methods in the presence of the indicated peptides at the indicated concentrations. The number of cells per field identified as fibrocytes by their spindle-shaped morphology was quantified. The Antennapedia Internalization Sequence alone was routinely used as the Control peptide; when tested scrambled CSD attached to the Antennapedia Internalization Sequence gave similar results. These data represent the average ± s.e.m. of six independent fields for each condition from five normal and five SSc donors. For comparisons of CSD and Cav-BC with Control peptide ** indicates p < 0.01 and * indicates p < 0.05. For Normal cells/Control peptide vs SSc cells/Control peptide p < 0.01.

Monocytes and monocyte-derived fibrocytes are believed to participate in lung fibrosis both as sources of cytokines and as the precursors of myofibroblasts [19-26]. Given that caveolin-1 levels regulate monocyte functions, we examined the effect of CSD and it subdomains on monocyte to fibrocyte differentiation in 12-day cultures (Figure 7). Both CSD and Cav-BC significantly inhibited differentiation at the routine 5 μM and even at the lowest concentration tested (0.01 μM). In contrast, control Antennapedia peptide and Cav-A had no effect at 5 μM.

**Figure 8:**
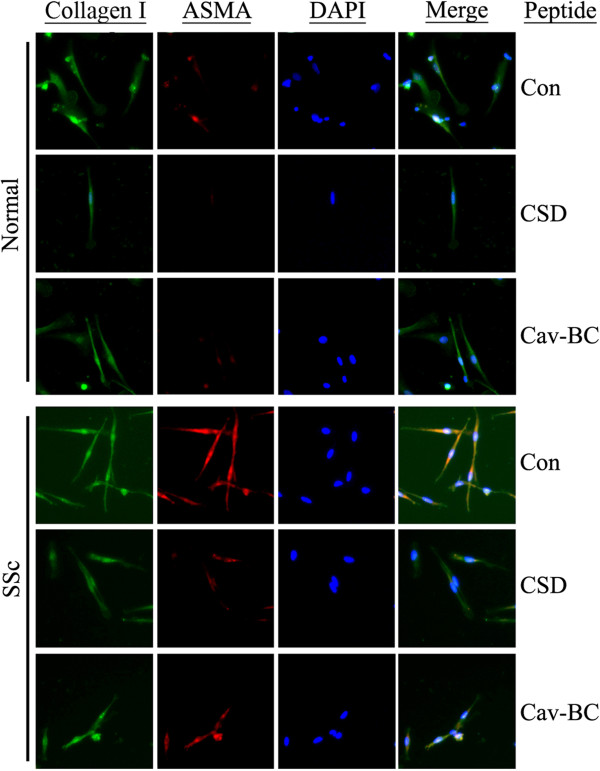
**ASMA staining is observed in SSc fibrocytes, but not normal fibrocytes, and is blocked by CSD and Cav-BC.** PBMC were incubated for fibrocyte differentiation in vitro on coverslips as described in the Methods in the presence of the indicated peptides at 0.1 μM, then stained for collagen I and ASMA and counterstained with the nuclear stain DAPI. The Antennapedia Internalization Sequence alone was routinely used as the Control peptide; when tested scrambled CSD attached to the Antennapedia Internalization Sequence gave similar results. Note the increased number of fibrocytes in SSc cultures, the inhibition of fibrocyte differentiation by CSD and Cav-BC in both SSc and normal cultures, the expression of ASMA in SSc fibrocytes but not in normal fibrocytes, the inhibition of ASMA expression by CSD and Cav-BC, and the similar levels of collagen I expression in fibrocytes in all cases. Similar results were obtained in four independent experiments.

When 12-day cultures were stained for collagen I (Figure 8), we observed a similar level of staining in normal and SSc fibrocytes which was not decreased by CSD or Cav-BC treatment (even though these treatments did decrease the number of fibrocytes present). However, when the cultures were stained for ASMA, we observed a high level of staining in SSc fibrocytes, but not in normal fibrocytes, and this accumulation of ASMA in SSc fibrocytes was almost completely blocked by CSD or Cav-BC. During a 21-day culture, as was shown previously [24,27], ASMA could be detected in normal fibrocyte cultures (data not shown), indicating that by the criterion of ASMA expression, SSc monocytes differentiate into fibrocytes more rapidly than do normal monocytes. The combined results highlight Cav-BC as an excellent candidate to be a subdomain of CSD active in the amelioration of lung fibrosis in vivo because it inhibits profibrotic features of monocytes as well as their migration and differentiation into fibrocytes.

### Dose-dependent effects of csd and subdomains on monocyte migration

**Figure 9:**
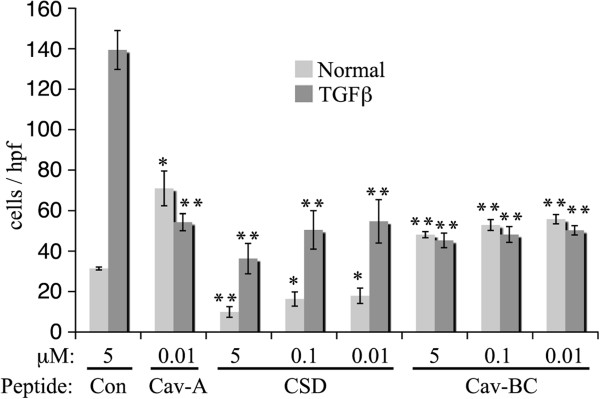
**Dose dependence of effects of CSD and its subdomains on the migration in vitro of Normal monocytes with and without TGFβ activation.** The migration toward CXCL12 of Normal monocytes with and without TGFβ activation was quantified as described in the Methods in the presence of the indicated concentration of CSD and its subdomains. The Antennapedia Internalization Sequence alone was routinely used as the Control peptide; when tested scrambled CSD attached to the Antennapedia Internalization Sequence gave similar results. The results represent the average ± s.e.m of four independent experiments. For comparisons of CSD, Cav-A, and Cav-BC with Control peptide ** indicates p < 0.01 and * indicates p < 0.05. For Normal cells/Control peptide vs TGFβ cells/Control peptide p < 0.001.

Given the Results highlighting the functional importance of Cav-A and Cav-BC, we examined the dose-dependence of CSD, Cav-A, and Cav-BC on the migration of normal monocytes with and without TGFβ treatment (Figure 9). Even when diluted an additional 500-fold (to 0.01 μM), CSD, Cav-A, and Cav-BC strongly inhibited the migration of TGFβ-activated normal monocytes. For unactivated normal monocytes, throughout the dose curve CSD inhibited migration while Cav-A promoted migration and Cav-BC slightly promoted migration.

### CSD and its subdomains differ in their ability to inhibit collagen I and ASMA expression and MEK/ERK signaling in NLF and SLF

**Figure 10:**
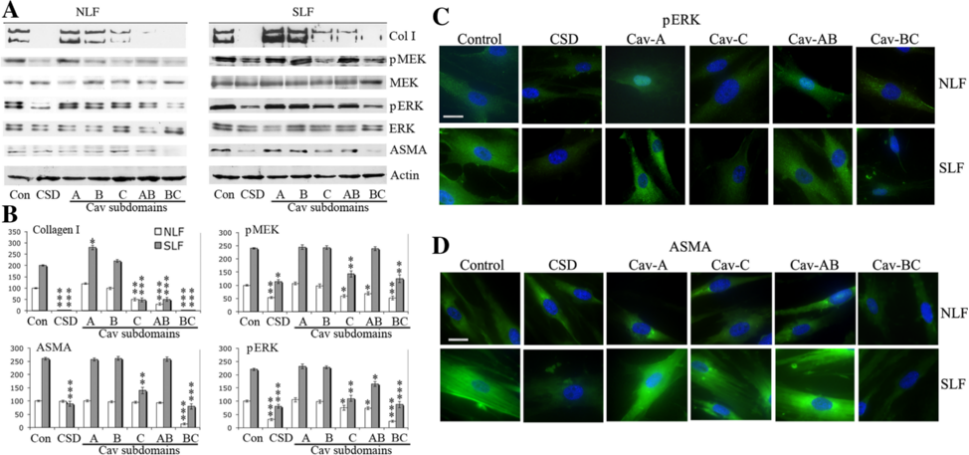
**Inhibition of collagen I and ASMA expression and MEK/ERK activation in fibroblasts by CSD and its subdomains.** Serum-starved NLF and SLF were incubated for an additional 6 h in fresh serum-free medium containing 5 μM of either CSD, Control peptide, or the indicated subdomain of CSD. The Antennapedia Internalization Sequence alone was routinely used as the Control peptide; when tested scrambled CSD attached to the Antennapedia Internalization Sequence gave similar results. The levels of collagen I in the culture medium and of pMEK, MEK, pERK, ERK, ASMA, and actin (loading control) in the cell layer were determined by Western blotting as described in the Methods **(A)**. **(B)** Densitometric quantification combining data from three experiments similar to **A** performed with three independent pairs of NLF and SLF. For comparisons of CSD and related peptides with Control peptide*** indicates p < 0.001, ** indicates p < 0.01, and * indicates p < 0.05. For NLF/Control peptide vs SLF/Control peptide for each chart in **(B)** p < 0.01. Immunofluorescent detection of effects of CSD and its subdomains on pERK **(C)** and ASMA **(D)** expression in NLF and SLF. Cells were cultured on coverslips under the conditions described above, then fixed and stained to detect the indicated proteins. Nuclei were counterstained with DAPI.

As in our previous studies, CSD inhibited collagen I expression in both NLF and SLF, but inhibited ASMA expression only in SLF. Cav-C, Cav-AB, and Cav-BC inhibited collagen I expression in both cell types (Figure 10AB) with Cav-BC being most effective, but had different effects on ASMA expression. In particular, Cav-C was similar to CSD in inhibiting ASMA expression in only SLF, Cav-BC inhibited ASMA expression in both cell types, while Cav-AB did not affect either cell type (Figure 10AB). Cav-A and Cav-B did not inhibit collagen I or ASMA expression. In fact, Cav-A clearly increased collagen I expression in SLF and slightly increased collagen I expression in NLF. As we previously demonstrated that MEK/ERK signaling regulates collagen I and ASMA expression [2,7], we determined the effect of CSD subdomains on the activation of these kinases. Cav-BC (like CSD) inhibited MEK and ERK activation in both cell types (Figure 10AB). Cav-C was somewhat less effective but still inhibited MEK and ERK in both cell types. Cav-AB slightly inhibited MEK in NLF and ERK in both NLF and SLF, while Cav-A and Cav-B were inactive.

To validate these observations and to learn more about the distribution of ASMA and activated ERK, we examined their expression and distribution by fluorescent microscopy (Figure 10CD). For ASMA (Figure 10C), as in Figure 10AB, CSD inhibited its expression only in SLF while Cav-BC inhibited its expression in both cell types. For activated ERK (Figure 10D), as in Figure 10AB, inhibition of expression by CSD, Cav-BC, and Cav-C was apparent. The slight inhibition observed in Figure 10AB for Cav-AB could not be detected by fluorescent microscopy. In addition, fluorescent microscopy revealed a change in subcellular distribution induced by Cav-A and, to a lesser extent, Cav-AB. Cav-A and Cav-AB caused activated ERK to translocate to the nucleus in NLF, but not in SLF.

**Figure 11:**
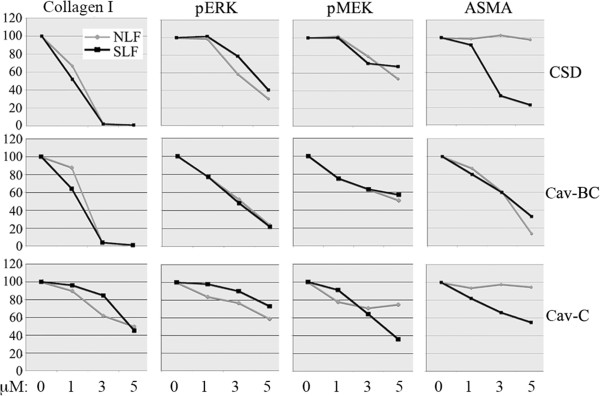
**Dose-dependence of inhibition of collagen I and ASMA expression and MEK/ERK activation in fibroblasts by CSD and its subdomains.** NLF and SLF were cultured as described in Figure 8 with 0, 1, 3, or 5 μM of the indicated peptides. Collagen I in the medium and pERK, ph-MEK, ASMA and actin (loading control) in the cell layer were detected by Western blotting. Blots were quantified densitometrically. The levels of collagen I, pERK, pMEK, and ASMA in cells treated with no peptide (divided by the level of actin) were set to 100 arbitrary units. The data presented are the average of the densitometric quantification of three independent experiments.

To further study the effects of CSD, Cav-BC, and Cav-C, we determined the dose-dependence of their effects on the expression of collagen I, ASMA, activated ERK, and activated MEK in NLF and SLF (Figure 11). In general, the effects that we observed using 5 μM peptide (Figure 10) were almost absent at 1 μM. These experiments validated the observation that CSD and Cav-C block ASMA expression in SLF but not in NLF because this effect was observed in cells treated with both 3 and 5 μM peptide (Figure 11). In contrast, Cav-BC inhibited ASMA expression similarly in both cell types at all concentrations tested.

**Figure 12:**
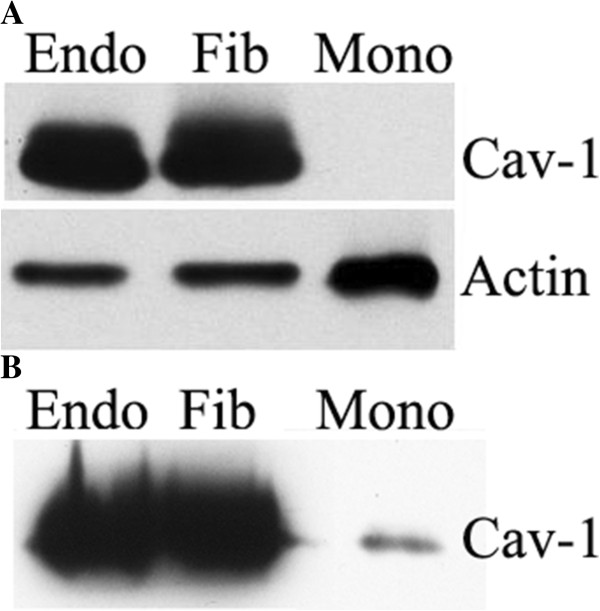
**Monocytes contain little caveolin-1 compared to fibroblasts and endothelial cells.** Human umbilical vein endothelial cells (Endo), NLF (Fib), and Normal monocytes (Mono) were extracted using SDS-PAGE sample buffer. **(A)** 10 μg each Endo and Fib extract and 30 μg Mono extract were Western blotted for caveolin-1 and actin (loading control). Similar results were obtained when GAPDH was used as the loading control (not shown). **(B)** Three times as much of each sample was loaded to allow the detection of caveolin-1 in Mono.

Given that CSD and related peptides must be used at 3 μM to affect fibroblast function but strongly affect monocyte function at 0.01 μM, we hypothesized that this effect results from a much lower caveolin-1 concentration in monocytes than in fibroblasts. Thus proportionally lower concentrations of peptide would be needed to reverse the effects of low caveolin-1 in SSc monocytes compared to SSc fibroblasts. To test this hypothesis, we compared the levels of caveolin-1 in normal monocytes, fibroblasts, and endothelial cells. Endothelial cells were included because the literature [12] shows that, like fibroblasts, micromolar CSD is required to affect the function of these cells. As predicted, the level of caveolin-1 is far less in monocytes than in either fibroblasts or endothelial cells (Figure 12), strongly supporting the idea that the extreme sensitivity of monocytes to CSD is due to the low baseline concentration of caveolin-1 in these cells.

### Specific amino acids involved in the function of CSD

Alanine screening revealed that amino acids 90 to 92 of caveolin-1 (particularly 92) are critical amino acids in CSD in the regulation of eNOS-dependent NO release from endothelial cells (Bernatchez, 2005). To determine whether these same amino acids are critical in the ability of CSD to regulate monocyte migration and differentiation and to regulate collagen I, pERK, pMEK, and ASMA expression in fibroblasts, we compared the activities of CSD and the mutated versions of CSD described as 92A and 90-92A.

**Figure 13:**
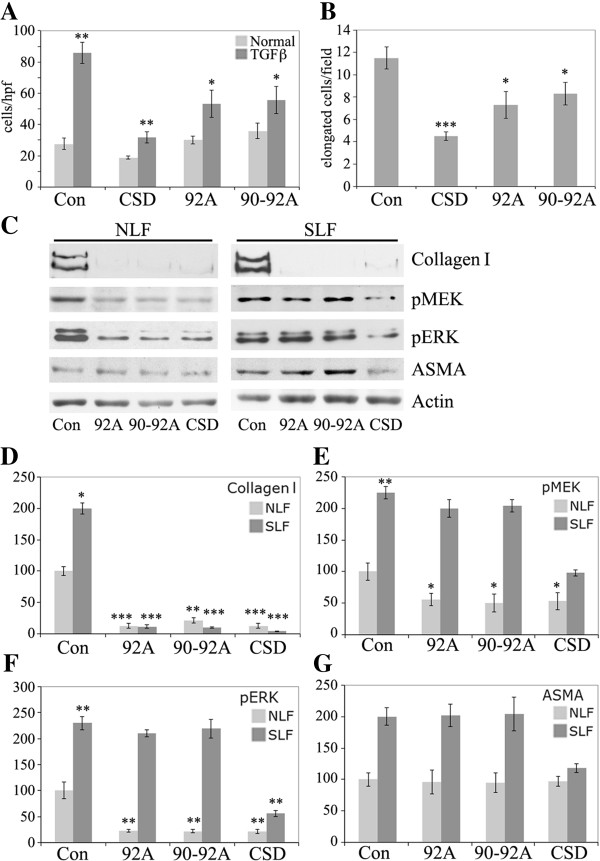
**Effects of mutated CSD on monocyte and fibroblast functions.** The activity of CSD and mutated forms (92A and 90-92A) were compared. **(A**,**B**) Monocytes. **(C**-**F)** Fibroblasts. **(A)** Monocyte migration experiments were performed with Normal monocytes and TGFβ-treated Normal monocytes and the indicated peptides (0.1 μM) as described in the Methods. Indication of statistical significance for TGFβ-treated Normal monocytes with Control peptide is versus Normal monocytes with Control peptide. Indications of statistical significance for TGFβ-treated Normal monocytes treated with CSD, 92A, or 90-92A is versus TGFβ-treated Normal monocytes treated with Control peptide. **(B)** Fibrocyte differentiation experiments were performed with Normal monocytes and the indicated peptides (0.1 μM) as described in the Methods. **(C)** The levels of collagen I in the culture medium and of pMEK, pERK, ASMA, and actin (loading control) in the cell layer were determined by Western blotting as described in the Methods using NLF and SLF treated with the indicated peptides as described in the legend to Figure 10. **(D**-**G)** Densitometric quantification combining data from three experiments similar to **(C)** performed with three independent pairs of NLF and SLF. **(D)** Collagen I. **(E)** pMEK. **(F)** pERK. **(G)** ASMA. Indications of statistical significance for SLF plus Control peptide are versus NLF plus Control peptide. Indications of statistical significance for NLF plus 92A, 90-92A, or CSD are versus NLF plus Control peptide. Indications of statistical significance for SLF plus 92A, 90-92A, or CSD are versus SLF plus Control peptide.

Compared to CSD, both 92A and 90-92A were partially active in their ability to inhibit the migration of TGFβ-treated Normal monocytes and in their ability to inhibit the differentiation of Normal monocytes into fibrocytes (Figure 13AB). Although CSD inhibits the migration of Normal monocytes that had not been treated with TGFβ, 92A and 90-92A had no effect on these poorly migrating cells.

A range of outcomes were observed with fibroblasts depending on the target protein and whether NLF or SLF were being studied (Figure 13C-G). 92A and 90-92A were fully as active as CSD in inhibiting collagen I expression in both NLF and SLF and in inhibiting pMEK and pERK in NLF. In contrast, 92A and 90-92A were inactive compared to CSD in inhibiting pMEK, pERK, and ASMA expression in SLF. None of CSD, 92A, or 90-92A inhibited ASMA expression in NLF (i.e. cells whose baseline expression of ASMA is low). Thus, just as the relative activities of Cav-A, Cav-B, Cav-C, Cav-AB, and Cav-BC are context dependent, the importance of amino acids 90 to 92 in the regulation of cell behavior is also context dependent in that it depends on the cell type and readout being studied.

## Discussion

In this study, we have examined cellular mechanisms involved in lung fibrosis that are affected when the deficiency of caveolin-1 in fibrotic lung tissue is reversed using specific subdomains of the CSD peptide and mutated versions of the CSD peptide. These critical cellular mechanisms are the migration of monocytes into damaged tissue and their differentiation into fibrocytes and the expression of collagen I by fibroblasts. Studies on downstream molecular mechanisms through which these versions of CSD mediate their effects are also presented. More detailed studies on molecular mechanism are underway.

We previously showed that monocytes and fibroblasts from SSc patients are deficient in caveolin-1, and that treatment of these cells with the CSD peptide compensates for this deficiency thereby reversing a number of pathological features of these cells. Our goal in this study was to determine whether different subdomains of CSD have different abilities to regulate parameters associated with fibrosis in monocytes and fibroblasts with the idea that a particular subdomain might be a more effective treatment for SSc than full-length CSD. Our results are summarized in Table 4.

**Table 4:** Differential effects of CSD subdomains on monocyte CXCR4 and F-actin expression

**Cells**	**Peptide**	**CXCR4**	**Statistical significance**	**F-actin**	**Statistical significance**
Normal	Control	23.2 ± 0.8		32.1 ± 2.6	
Normal	CSD	20.6 ± 1.8		26.8 ± 1.7	
Normal	Cav-A	40.6 ± 1.9	p < 0.01 vs Normal/Control	85.8 ± 3.6	p < 0.01 vs Normal/Control
Normal	Cav-B	34.6 ± 1.2	p < 0.01 vs Normal/Control	93.9 ± 2.2	p < 0.01 vs Normal/Control
Normal	Cav-C	28.7 ± 0.8	p < 0.01 vs Normal/Control	73.7 ± 5.0	p < 0.01 vs Normal/Control
Normal	Cav-AB	23.9 ± 0.8		42.0 ± 2.1	p < 0.05 vs Normal/Control
Normal	Cav-BC	20.9 ± 0.7		34.5 ± 1.4	
Normal + TGFβ	Control	50.1 ± 2.6	p < 0.01 vs Normal/Control	40.7 ± 1.4	p < 0.05 vs Normal/Control
Normal + TGFβ	CSD	23.7 ± 0.8	p < 0.01 vs Normal + TGFβ/Control	35.4 ± 2.0	p < 0.05 vs Normal + TGFβ/Control
Normal + TGFβ	Cav-A	21.9 ± 0.6	p < 0.01 vs Normal + TGFβ/Control	49.0 ± 3.6	
Normal + TGFβ	Cav-B	32.4 ± 1.1	p < 0.01 vs Normal + TGFβ/Control	75.2 ± 3.7	p < 0.01 vs Normal + TGFβ/Control
Normal + TGFβ	Cav-C	27.6 ± 0.8	p < 0.01 vs Normal + TGFβ/Control	54.7 ± 4.5	p < 0.01 vs Normal + TGFβ/Control
Normal + TGFβ	Cav-AB	22.3 ± 0.6	p < 0.01 vs Normal + TGFβ/Control	47.5 ± 4.7	
Normal + TGFβ	Cav-BC	20.4 ± 0.8	p < 0.01 vs Normal + TGFβ/Control	38.7 ± 1.6	
SSc	Control	43.8 ± 2.6	p < 0.01 vs Normal/Control	129.2 ± 11.9	p < 0.01 vs Normal/Control
SSc	CSD	27.6 ± 1.4	p < 0.01 vs SSc/Control	58.3 ± 2.8	p < 0.01 vs SSc/Control
SSc	Cav-A	51.4 ± 2.6		138.1 ± 7.3	
SSc	Cav-B	32.6 ± 1.6	p < 0.01 vs SSc/Control	60.9 ± 5.7	p < 0.01 vs SSc/Control
SSc	Cav-C	31.5 ± 1.5	p < 0.01 vs SSc/Control	100.0 ± 8.6	
SSc	Cav-AB	30.9 ± 2.3	p < 0.01 vs SSc/Control	79.5 ± 5.1	p < 0.01 vs SSc/Control
SSc	Cav-BC	27.7 ± 0.8	p < 0.01 vs SSc/Control	92.9 ± 4.5	p < 0.05 vs SSc/Control

Image analyses of Figures 5 and 6 are quantified in terms of average fluorescence intensity ± s.e.m. and the statistical significance of the indicated comparisons determined using Students’ t test.

Migration toward CXCL12 is enhanced several-fold in TGFβ-treated normal monocytes and even more in SSc monocytes [17]. While CSD inhibits migration in all three cell populations and at very low doses, the subdomains have different effects (Table 4). Cav-BC also strongly inhibits at very low doses the migration of cells that migrate well (TGFβ-treated and SSc monocytes). Lesser inhibition of migration in TGFβ-treated monocytes was obtained with the other subdomains while Cav-A slightly enhanced SSc monocyte migration. Cav-A, and other subdomains to a lesser extent, also enhanced the migration of normal monocytes (i.e. not TGFβ-treated).

Because TGFβ treatment strongly enhanced monocyte migration, we also examined canonical TGFβ signaling via Smad 2/3. In many cases, the effects of CSD and its subdomains on monocyte migration and Smad 2/3 activation were similar. For example, CSD and Cav-BC strongly inhibit the migration of monocytes that migrate well (i.e. TGFβ-treated Normal monocytes and SSc monocytes) and also strongly inhibit Smad 2/3 activation in these cells. Likewise, the enhancement of migration that occurs in Normal monocytes treated with Cav-A was accompanied by an enhancement of Smad 2/3 activation in these cells. In other cases, the effects of subdomains on migration and Smad 2/3 activation were not similar. For example, several subdomains inhibited migration in TGFβ-treated Normal monocytes without affecting Smad 2/3 activation; several subdomains enhanced migration in Normal monocytes while inhibiting Smad 2/3 activation. An additional striking observation revealed by these studies is that while the levels of activated Smad 2/3 are very high in SSc monocytes compared to Normal monocytes, the enhancement in the level of total Smad 2/3 is even more pronounced. In summary, we find that while CSD and its subdomains can affect TGFβ signaling via pSmad 2/3, it is likely that their effects on TGFβ signaling combine with their well-known effects on other signaling cascades to regulate monocyte migration.

We report here the novel observation that the differentiation of SSc monocytes into spindle-shaped, ASMA-positive, collagen I-positive fibrocytes is enhanced compared to normal monocytes (Figure 9). In both cell types CSD and Cav-BC inhibit monocyte differentiation at very low doses, while Cav-A has no effect even at a high dose (Figure 9, Table 4). In summary, Cav-BC and Cav-A are of particular interest. Cav-BC, like CSD, inhibits the pathological hypermigration of TGFβ-treated and SSc monocytes and the differentiation into fibrocytes of both Normal and SSc monocytes. Unlike CSD, Cav-BC does not inhibit the migration of Normal monocytes. Cav-A enhances the migration of both Normal and SSc monocytes although it partially inhibits the migration of TGFβ-treated monocytes and has no effect on differentiation.

We previously reported that collagen I expression and MEK/ERK signaling in fibroblasts (both NLF and SLF) is inhibited by CSD but that ASMA expression is only inhibited by CSD in cells expressing it at high levels (i.e. SLF) [2]. Here we report that Cav-BC, like CSD, inhibits collagen I expression and MEK/ERK signaling in both NLF and SLF. However, unlike CSD, Cav-BC also inhibits ASMA expression in both cell types. The function of Cav-A in fibroblasts is also noteworthy, causing an increase in collagen I expression in SLF, a slight increase in collagen I in NLF, and the nuclear translocation of ERK in NLF. Thus, in both monocytes and fibroblasts Cav-BC is the subdomain most similar in function to CSD while Cav-A has a variety of distinct, potentially pro-fibrotic, functions. In addition to the differences between the effect of each peptide on the expression of collagen I and ASMA and the differences between NLF and SLF in their response to a given peptide, our results were also very different than those of Bernatchez et al. [11] who examined the sensitivity of eNOS activity in endothelial cells to these same peptides. As shown in Table 4, eNOS activity was most strongly inhibited by intact CSD and Cav-B and was also inhibited by Cav-AB and Cav-BC. Therefore, Table 4 demonstrates that the ability of each peptide to regulate the expression of a particular target protein depends on the target protein and on the cell type being studied.

To further explore differences between particular cell types and particular target proteins in terms of how they are affected by different versions of CSD, we studied two mutated versions of CSD (92A and 90-92A) that were previously shown to be totally ineffective in inhibiting eNOS-mediated generation of NO in endothelial cells [11]. In contrast, 92A and 90-92A were partially active in inhibiting monocyte migration or differentiation into fibrocytes. Moreover, 92A and 90-92A were essentially as effective as CSD in inhibiting Collagen I expression in NLF and SLF and in inhibiting MEK and ERK activation in NLF. In contrast, they were almost inactive in inhibiting MEK and ERK activation and ASMA expression in SLF. These observations further support the conclusion that the ability of versions of CSD to regulate the expression of a particular target protein depends on the target protein and on the cell type being studied.

Table 3 allows us to speculate on whether CSD and its subdomains regulate monocyte migration through their effects on CXCR4 or F-actin expression. Our observations can be summarized as: 1) The enhanced migration of TGFβ-treated normal monocytes is correlated primarily with increased CXCR4 expression while the enhanced migration of SSc monocytes is correlated both with enhanced CXCR4 and F-actin expression; 2) For each type of monocyte (Normal, Normal + TGFβ, SSc), CSD and it subdomains had essentially parallel effects on migration and CXCR4 expression; 3) In cells that migrate well (Normal + TGFβ and SSc monocytes), Cav-BC and CSD are the most effective inhibitors of migration due to their inhibition of CXCR4 expression. In contrast, TGFβ-treated and SSc monocytes differ greatly in that Cav-A inhibits CXCR4 expression and migration in TGFβ-treated monocytes while having no effect on these parameters in SSc monocytes; 4) In cells that do not migrate well (Normal monocytes), Cav-A promotes migration due to its positive effects on both CXCR4 and F-actin levels. 5) A comparison of the data obtained with various combinations of cell type and peptide makes it clear that other factors besides CXCR4 and F-actin expression control migration. For example, levels of CXCR4 and F-actin in SSc monocytes in the presence of Cav-BC are similar to their levels in Normal monocytes in the presence of Cav-B yet the SSc monocytes exhibit four-fold higher migration than the Normal monocytes. Thus, the enhanced migration of SSc monocytes compared to Normal monocytes must involve more differences between these cell types than simply their CXCR4 and F-actin levels.

These studies have revealed that monocytes are much more sensitive to CSD and its subdomains than are fibroblasts. In studies using fibroblasts [2] and endothelial cells [11], 5 to 10 μM CSD was used to compensate for a loss of caveolin-1. The current studies demonstrate that for fibroblasts this level of CSD and subdomains is required, because little or no effect is observed at 1 μM. In contrast, in experiments using monocytes, we report that CSD and Cav-BC are as active at 0.01 μM as they are at 5 μM. These observations raise the possibility that monocytes are more sensitive to CSD and its subdomains than are fibroblasts and endothelial cells because the baseline level of caveolin-1 in monocytes is much lower than in these other cell types. Indeed, we have demonstrated in Figure 12 that this is the case. This further suggests that while the use of CSD or its subdomains may have a therapeutic effect in human patients by reversing the profibrotic and proinflammatory effects of low caveolin-1 in monocytes and fibrocytes, CSD and subdomains may not have side effects in fibroblasts and endothelial cells because the small increase in caveolin-1 function in these cells that already contain caveolin-1 at high levels is not likely to have an appreciable effect on their function.

Although many authors have proposed that TGFβ is the major cytokine responsible for the pathology of SSc and have used TGFβ-treated cells as a model for SSc, the current results demonstrate both differences and similarities between SSc and Normal + TGFβ monocytes (Tables 3, 4, and 5). These observations suggest that pathways other than TGFβ are involved in the pathology of SSc and that the sensitivity of these pathways to CSD and to its subdomains differ. Therefore, it is not surprising, for example, that Cav-A inhibits the migration of Normal + TGFβ monocytes, but slightly enhances the migration of Normal and SSc monocytes.

**Table 5:** Differential effects of CSD subdomains on monocyte migration, fibrocyte differentiation, and fibroblast collagen and ASMA expression

**Peptide**	**Monoycte migration**			**Fibrocyte differentiation**		**Protein expression in fibroblasts and endothelial cells**				
	**Normal**	**TGFβ**	**SSc**	**Normal**	**SSc**	**Collagen NLF**	**Collagen SLF**	**ASMA NLF**	**ASMA SLF**	**eNOS End**
Control	20.1	99.9	303	30	54	100	200	100	260	
CSD	↓	↓↓	↓↓	↓	↓	↓↓	↓↓	No Effect	↓↓	↓
Cav-A	**↑↑**	↓	No Effect	No Effect	No Effect	No Effect	**↑**	No Effect	No Effect	No Effect
Cav-B	**↑**	↓	Not Done	Not Done	Not Done	No Effect	No Effect	No Effect	No Effect	↓
Cav-C	**↑**	↓	Not Done	Not Done	Not Done	↓	↓↓	No Effect	↓	No Effect
Cav-AB	**↑**	↓	Not Done	Not Done	Not Done	↓↓	↓↓	No Effect	No Effect	↓
Cav-BC	**↑**	↓↓	↓	↓	↓	↓↓	↓↓	↓↓	↓↓	↓

The Control Peptide row shows the baseline values for each cell type for Monocyte Migration (in Cells/hpf from Figure 2 and Table 3), for Fibrocyte Differentiation (in Elongated cells/field from Figure 7), and for Protein Expression in Fibroblasts (in Arbitrary Units from Figure 10). eNOS expression in Endothelial Cell data are from ref. [11]. The other rows show increases and decreases from these baseline levels caused by CSD and related peptides (**↑↑** = > 100% increase, **↑** = 35 to 100% increase, No Effect = 35% decrease to 35% increase, ↓ = 35 to 65% decrease, ↓↓ = 65 to 100% decrease.

## Conclusions

These studies have revealed Cav-BC to be an anti-inflammatory, anti-fibrotic subdomain of CSD that may be useful in treating fibrotic lung diseases in human patients. In contrast, Cav-A has certain pro-inflammatory, pro-fibrotic functions which may make it a useful treatment for other diseases such as wound healing. These studies have also revealed major differences between Normal monocytes activated with TGFβ and SSc monocytes that suggest that the pathology of this disease is more complex than simply hyperactivated TGFβ signaling. Future studies will expand upon the peptide-specific and cell type-specific differences in signal transduction already observed that must underlie these complex observations. Finally, we observed that monocytes are much more sensitive to CSD and its subdomains than are fibroblasts, suggesting that in vivo monocytes and fibrocytes will be selectively affected by CSD treatment without the treatment having significant side effects on other cell types.

## Abbreviations

ASMA: α-smooth muscle actin; CXCL12: C-X-C chemokine ligand 12; CXCR4: C-X-C Chemokine receptor type 4; eNOS: Endothelial Nitric Oxide Synthase; ERK: Extracellular signal-regulated kinases; ILD: Interstitial lung disease; IPF: Idiopathic pulmonary fibrosis; MEK: MAPK/ERK kinase; NO: Nitric oxide; SSc: Systemic sclerosis, scleroderma; TGFβ: Transforming growth factor β; PI: Propidium iodide.

## Competing interests

While none of the authors have received any financial benefit from this work, Drs. Hoffman and Tourkina are the Inventors on a use patent (# 8,058,227) issued to the Medical University of South Carolina on the caveolin-1 scaffolding domain peptide as a treatment for fibrotic diseases. Drs. Hoffman and Tourkina are also the founders of a company, FibroTherapeutics, Inc., which has an option to license this patent from MUSC for the purpose of developing a drug based on the caveolin-1 scaffolding domain peptide.

## Authors’ contributions

CR participated in study design, monocyte isolation and all studies on these cells, data interpretation, and manuscript preparation. SD and BP performed monocyte isolation and studies. MB participated in monocyte to fibrocyte differentiation studies and manuscript preparation. JO participated in data interpretation and manuscript preparation. AH performed immunocytochemical analyses on human cells. RPV participated in study design, data interpretation and manuscript preparation. JZ participated in immunocytochemical studies on monocytes. CMH analyzed patient demographics. RMS participated in data interpretation and manuscript preparation. SH participated in study design, data interpretation, manuscript preparation and performed statistical analyses. ET participated in study design, human lung fibroblasts studies, data interpretation, and manuscript preparation. All authors have read and approved the final manuscript.
